# Functional Outcome in Sarcomas Treated With Limb-Salvage Surgery or Amputation

**DOI:** 10.1080/13577149878118

**Published:** 1998-03

**Authors:** Rikke Johansen, Ole S. Nielsen, Johnny Keller

**Affiliations:** 1 Centre for Bone and Soft Tissue Sarcomas Aarhus University Hospital Denmark; 2 Department of Oncology Aarhus University Hospital Aarhus C DK-8000 Denmark

## Abstract

*Purpose.* In all patients treated at the Centre for Bone and Soft Tissue Sarcomas of Aarhus the functional outcome is prospectively evaluated by use of the Enneking system for the functional evaluation after surgical treatment of tumours of the musculoskeletal system. This system has been accepted by the Musculoskeletal Tumour Society and the International Symposium on Limb Salvage.

*Patients/methods.* In the present study the functional outcome after limb-salvage surgery (89 patients) and amputation (58 patients) was compared. In the limb-salvage group the treatment was surgery alone in 50% and surgery combined with either radiotherapy in 39% or chemotherapy in 11%. Inclusion criteria were: Deep seated extremity sarcomas, age >14 years, more than 1 year post-treatment follow-up time and alive at the end of the study. Median age was 49 years (range 14–88 years). Median tumour diameter was 8 cm (range 1–20 cm), median follow-up time was 4.8 years (range 1–11 years). Wilcoxon and ^χ2^-tests were used for statistical analyses.

*Results.* The two groups were comparable according to age, sex, size of tumour, type of tumour, location of tumour, as well as post-treatment follow-up time. The functional scores were significantly higher after limb-salvage surgery as compared to amputation, the median scores being 85 and 47, respectively (p<0.001). A similar difference was observed if the Enneking scores were subdivided into general health-related scores and extremity-related scores. No association was found between functional scores and the following factors by use of univariate analysis: size of tumour, radiation therapy, localization of tumour and surgical margin.

*Discussion.* We conclude that this study indicates that limb-salvage surgery is associated with a better functional outcome than that observed after amputation. However, whether this also indicates a difference in quality of life needs further studies.


*Sarcoma* (1998) **2**, 19–23

CARFAX

ORIGINAL ARTICLE

### Functional outcome in sarcomas treated with limb-salvage surgery or amputation

RIKKE JOHANSEN, OLE S. NIELSEN & JOHNNY KELLER

*Centre for Bone and Soft Tissue Sarcomas, Aarhus University Hospital, Denmark*

**Abstract**

*Purpose.* In all patients treated at the Centre for Bone and Soft Tissue Sarcomas of Aarhus the functional outcome is prospectively evaluated by use of the Enneking system for the functional evaluation after surgical treatment of tumours of the musculoskeletal system. This system has been accepted by the Musculoskeletal Tumour Society and the International Symposium on Limb Salvage.

*Patients/methods.* In the present study the functional outcome after limb-salvage surgery (89 patients) and amputation (58 patients) was compared. In the limb-salvage group the treatment was surgery alone in 50% and surgery combined with either radiotherapy in 39% or chemotherapy in 11%. Inclusion criteria were: Deep seated extremity sarcomas, age $>14$ years, more than 1 year post-treatment follow-up time and alive at the end of the study. Median age was 49 years (range 14–88 years). Median tumour diameter was 8 cm (range 1–20 cm), median follow-up time was 4.8 years (range 1–11 years). Wilcoxon and $\chi^2$-tests were used for statistical analyses.

*Results.* The two groups were comparable according to age, sex, size of tumour, type of tumour, location of tumour, as well as post-treatment follow-up time. The functional scores were significantly higher after limb-salvage surgery as compared to amputation, the median scores being 85 and 47, respectively ($p < 0.001$). A similar difference was observed if the Enneking scores were subdivided into general health-related scores and extremity-related scores. No association was found between functional scores and the following factors by use of univariate analysis: size of tumour, radiation therapy, localization of tumour and surgical margin.

*Discussion.* We conclude that this study indicates that limb-salvage surgery is associated with a better functional outcome than that observed after amputation. However, whether this also indicates a difference in quality of life needs further studies.

**Key words**: *functional outcome, limb-salvage surgery, amputation, Enneking system, bone tumours, soft tissue tumours.*

### Introduction

Previously, patients with extremity tumours were routinely treated with amputation. Within the last 15–20 years the preferred treatment has shifted, and today the majority of patients are offered limb-salvage surgery. Several factors have played a role in this shift of treatment strategy, the most important being: the development within the field of adjuvant therapies in the form of radiation therapy (soft tissue tumours) and chemotherapy (bone tumours), improved image diagnostics such as magnetic resonance imaging (MRI) and computed tomography (CT) as well as improved techniques of excision and reconstruction.[1,2]

Amputation is an extensive and often invalidating procedure, but in spite of this the patient can often be discharged and mobilized after a relatively short period of time. In contrast, limb-salvage surgery may cause long hospitalization and result in a not optimally functioning limb. Due to individual factors, such as age, social and personal relations as well as the nature of the tumour, it is important to be able to offer each patient an individual treatment. Since sarcoma is a rare disease and the patients should be offered a multi-disciplinary treatment, the management of these patients should only be performed in centres with expertise in treatment of sarcomas.[3,4]

In order to apply the most suitable treatment for each patient, it is of great importance to be able to evaluate a given treatment. In this perspective, a standardized, validity-tested system of evaluation of function is necessary. Several systems of evaluation have previously been used. However, these systems have focused on the function of the operated extremity rather than the general condition of the patient. Furthermore, these systems have often been based on the doctor's judgement, and studies

Correspondence to: O. S. Nielsen, Department of Oncology, Aarhus University Hospital, DK-8000 Aarhus C, Denmark. Fax: +45 89492550; E-mail: osn@onko.aau.dk.

1357-714X/98/040019-05 ©1998 Carfax Publishing Ltd confirming that the data obtained by these methods agree with that of the patient are lacking.

A system for functional evaluation following tumour surgery described by Enneking has been field tested in 1989 by the Musculoskeletal Tumour Society (MSTS) and adopted by the MSTS and the International Syrnposium on Limb-salvage.[1] The system is based on a questionnaire and a simple clinical test.

The aim of the present study was to investigate the functional outcome after amputation and limb-salvage surgery of sarcomas by use of the Enneking system. In addition, the influence of different factors on the functional results was examined.

### Patients and materials

For all patients surgically treated for sarcomas at the Centre for Soft Tissue and Bone Sarcomas at the University Hospital in Aarhus during the period 1983–1995, information concerning treatment and tumour have been registered. After treatment, all patients were regularly seen in the outpatient clinic for observation of possible recurrence. The post-operative functional outcome was prospectively examined by use of the Enneking system for functional evaluation, which consists of a questionnaire and a simple clinical test. The questionnaires were filled out independently by the patients, and the clinical test was carried out by the doctor present in the outpatient clinic. The system assigns numerical values (0–5) for each of six categories: pain, function and emotional acceptance in the upper and in the lower extremity, respectively. In addition, in the lower extremity, supports, walking as well as gait, and in the upper extremity, hand positioning, dexterity as well as lifting ability were assigned. To allow a comparison of results, numerical scores and percentage ratings were calculated. The system has been field tested, and is accepted and recommended by the MSTS.[1]

Patients were selected for the study based on the following criteria of inclusion: age 14 years or above; malignant, deeply localized extremity tumours; at least 1 year of post-treatment follow-up time; alive at the end of the study; amputation or limb-salvage surgery. The general principles for performing either amputation or limb-salvage surgery were as follows. In the first part of the period, compartmental resection or amputation were aimed at for highly malignant tumours. If this was not achievable due to the localization or dissemination of the tumour, resection with the largest possible margin followed by radiation therapy was carried out. Later, this principle was changed to combined surgery and radiotherapy in most patients.

The criteria of inclusion were fulfilled by 147 patients. The patients were divided in two groups according to the type of operation that was performed; limb-salvage surgery or amputation.

### *The limb-salvage surgery group*

Eighty-nine (46 females, 43 males) patients had limb-salvage surgery. Median age was 50 years (range 14–87 years). The median diameter of the tumours was 8 cm (range 1–20 cm). Twenty tumours were $<5$ cm in diameter and 69 were $\geqslant 5$ cm. Twenty-one patients had bone tumours and 68 soft tissue tumours. Twenty-seven tumours were localized in the upper extremity and 62 in the lower extremity. Two-thirds of the bone tumours were localized in tibia, humerus and femur, while more than half of the soft tissue tumours were localized in the thigh. Chondro- and osteosarcomas dominated in the bone tumours accounting for more than 90%. In the soft tissue tumours, liposarcomas and malignant fibrous histiocytoma (MFH) accounted for more than 50%.

The histopathogical grades of the tumours were determined on basis of microscopy of the removed tumour.[5] Thirty patients had grade I tumours, 20 grade II, 16 grade IIIA, 11 grade IIIB and 12 patients had malignant tumours whose histological grades could not be described more precisely. Forty-five patients in this group received limb-salvage surgery as the only treatment, while 34 patients were treated with combined limb-salvage surgery and radiation therapy, nine patients with combined limb-salvage surgery and chemotherapy, and one patient with limb-salvage surgery as well as radiation therapy and chemotherapy. In general, chemotherapy was given to patients with bone tumours, and radiation therapy to patients with soft tissue tumours. In this context, the term 'combined' means that radiation therapy or chemotherapy were given at some point in the course of treatment, not necessarily adjuvant to the primary surgical treatment. Yet for 91% of the patients the radiation therapy was given adjuvant to the primary surgical treatment.

### *The amputation group*

Fifty-eight patients were treated with amputation (40 males, 18 females). Median-age was 47 years (range 14–88 years). The median tumour size was 8 cm (range 1–20 cm). Ten tumours were $<5$ cm in diameter and 48 were $\geqslant 5$ cm.

Twenty-five patients had bone tumours and 33 patients had soft tissue tumours. Forty-two (72%) tumours were localized to the lower extremities and 16 (28%) to the upper extremities. Two-thirds of the bone tumours were localized in the femur and the tibia, while more than the half of the soft tissue tumours were localized in the thigh, knee and the lower leg. Among the bone tumours, osteo- and chondrosarcomas dominated. MFH comprised one-third of the soft tissue sarcomas.

The distribution of the tumour grades was as follows: six patients had grade I tumours, seven grade II, 23 grade IIIA, 12 grade IIIB and 10

**Table 1.** *The comparability between the limb-salvage and the amputation group*

| Parameter | Limb-salvage surgery | Amputation | $p$-Value |
|---|---|---|---|
| Number of patients | 89 | 58 | |
| Age (years)* | 50 (14–87) | 46 (14–88) | 0.06 |
| Sex (F/M) | 46/43 | 18/40 | 0.08 |
| Upper/lower extremity | 27/62 | 16/42 | 0.99 |
| Soft tissue/bone | 21/68 | 25/33 | 0.30 |
| Size (cm)* | 8 (1–20) | 8 (1–20) | 0.57 |
| Follow-up time (year)* | 4.2 (1–10) | 5.2 (1–11) | 0.11 |

*Median (range).

**Table 2.** *The overall Enneking functional score as well as the general health, and the upper and lower extremity-related scores in the limb-salvage and amputation group*

| Median functional score | Limb-salvage surgery* | Amputation* | $p$-Value |
|---|---|---|---|
| Overall Enneking | 85 (10–100) | 47 (13–87) | $<0.001$ |
| General health related | 80 (20–100) | 60 (13–93) | $<0.01$ |
| Upper extremity related | 87 (7–100) | 37 (7–87) | $<0.01$ |
| Lower extremity related | 93 (27–100) | 33 (7–87) | $<0.01$ |

*Median (range).

patients had malignant tumours where the histological grade could not be described more precisely.

Forty-one patients in this group received surgery as the only treatment. Four patients were treated with combined surgery and radiation therapy, while 13 patients were treated with combined surgery and chemotherapy.

### *Statistics*

To compare the post-operative functional results between the two groups, Wilcoxon's range-sum test was used. The $\chi^2$-test was used to test if two groups were comparable according to patient characteristics.

### Results

The two groups were comparable according to age, sex, time of observation and tumour size, including the distribution of small and large tumours ($<$ or $\geq 5$ cm). Also the distribution of bone and soft tissue tumours as well as the distribution of tumours localized in the upper and lower extremities were comparable in the two groups (Table 1).

Patients treated with limb-salvage surgery ($n = 89$) had a significantly higher functional score compared to the group of patients undergoing amputation ($n = 58$) (Table 2). The functional median score following limb-salvage surgery was 85 (range 10–100) as compared to 47 (range 13–87) after amputation ($p < 0.001$). The Enneking system for functional evaluation can be subdivided into a general health-related score and an upper and a lower extremity-related score. We found that both the general health score and the extremity-related scores were significantly higher in the limb-salvage group compared to the amputation group (Table 2).

Since the outcome of low- and high-grade tumours, might differ, the analysis was also performed after excluding grade I tumours in both the groups. However, also in this group of high-grade tumours, we found a significantly higher functional score in the group of patients treated with limb-salvage surgery ($n = 59$) compared to the group of patients operated with amputation ($n = 52$). The median functional score following limb-salvage surgery was 83 (range 10–100) compared to 47 (range 13–87) after amputation ($p < 0.001$).

The influence of different factors on the functional score was tested. In neither the limb-salvage group nor the amputation group did the localization of tumour result in a difference in functional scores.

For the limb-salvage group the functional score according to the type of tumour (soft tissue or bone) was examined (Table 3). Patients with soft tissue tumours ($n = 68$) scored significantly higher than patients with bone tumours ($n = 21$). An endoprothesis was used in 10/21 patients who had bone tumours. Those patients had a lower functional score compared to patients with soft tissue tumours. No significant difference in functional score was found between patients with soft tissue tumours

**Table 3.** *The Enneking functional score according to the type of tumour (soft tissue or bone) in the limb-salvage group*

| Tumour type | Endoprothesis | Median functional score** |
|---|---|---|
| Soft tissue | No | 87 (23–100)* |
| Bone | Yes | 65 (10–89)* |
| Bone | No | 73 (20–100) |

*Difference significant ($p < 0.05$).

**Median (range).

**Table 4.** *The median functional scores among patients treated with limb-salvage surgery and divided into subgroups according to the surgical margins obtained*

| Parameter | Surgical margin: Intralesional | Marginal | Wide | Compartmental | $p$-Value |
|---|---|---|---|---|---|
| Number of patients | 5 | 19 | 49 | 15 | |
| Age (years) | 56 | 54 | 50 | 59 | 0.14 |
| Size of tumour (cm) | 11 | 6 | 7 | 9 | 0.13 |
| Grade (low/high) | 4/1 | 5/14 | 16/33 | 5/10 | 0.15* |
| Functional scores** | 93 (23–93) | 87 (30–100) | 85 (30–100) | 73 (40–100) | 0.95 |

*In the calculation of a $p$-value the intralesional and marginal group were combined.
**Median (range).

and patients with bone tumours having limb-salvage surgery without the use of an endoprothesis.

In the group of patients treated with limb-salvage surgery, there was a tendency to a better functional score among patients with tumours $<5$ cm ($n=20$) compared to patients with tumours $>5$ cm ($n=69$), the median score being 90 (range 60–100) and 82 (range 10–100), respectively ($p=0.07$).

In the limb-salvage group, surgery was combined to radiation therapy in 34/89 patients. No significant correlation was found between radiation therapy and the functional score, the median score being 87 (range 23–100) for patients receiving radiation therapy and 83 (range 10–100) for patients who were not treated with radiation therapy.

Table 4 shows the functional scores in relation to the surgical margin achieved after performing limb-salvage surgery. The patients were divided into four subgroups according to surgical stages.[6,7] The subgroups were comparable according to age, size of tumour as well as the distribution of low (grade I) and high-grade tumours (grade II–III). No significant difference in functional scores was found between the four subgroups treated with differently surgical margins. Also there was no significant difference in functional score if patients treated with intralesional, marginal and wide margins were combined into one group and compared to patients treated with compartmental margin.

### Discussion

The present study showed a higher functional score in the limb-salvage group compared to the amputation group using the Enneking functional system. The same result was found when studying the group of high-grade tumours only. Several studies have evaluated the functional outcome following limb-salvage procedures,[8–13] but to our knowledge only a single study has compared the functional results after limb-salvage and amputation with use of the Enneking system of functional evaluation. Rougraff *et al.*[14] compared the functional results among patients with lower extremity osteosarcomas using the Enneking system for functional evaluation, and similarly to our study they observed a better functional outcome following limb-salvage. On the other hand, they failed to find any difference between the groups when analyzing only the scores related to the patient's general condition—a difference which had been observed in other studies. Sugarbaker *et al.*[15] demonstrated that there was no difference in psychosocial adjustment to illness or in quality of life between patients who were treated with limb-salvage surgery and patients treated with amputation. Similarly, Weddington *et al.*[16] were unable to detect a significant difference in psychosocial outcome between patients who underwent amputation and limb-salvage.

In the present study, both the general health-related scores and the extremity-related scores were significantly higher in the limb-salvage group compared to the amputation group. In the limb-salvage group the general health-related score and the extremity-related scores were of equal size. In contrast, the general health-related score was higher than both the upper and the lower extremity-related scores in the amputation group. This suggests that the low functional score in the amputation group is especially due to a functional reduction of the operated extremity rather than an impairment of the patients' general condition. This may support the findings of other authors[15,16] who also failed to demonstrate any difference in quality of life after amputation and limb-salvage. Several studies[8,13] have reported that radiation therapy may have a great impact on the functional results. At least in the present study, we were unable to demonstrate that radiation therapy had any effect on the functional outcome.

Among patients who underwent limb-salvage, our results showed a significant lower functional score in patients with soft tissue tumours than in patients with bone tumours. The fact that half of the patients with bone tumours had an endoprothesis could explain this observation, because those were the patients that actually had a low score compared to patients with soft tissue tumours. In the group of patients with bone tumours that did not have an endoprothesis, there was no difference in functional score compared to those patients with soft tissue tumours.

In the present study, a number of subanalyses of the influence of different factors on the functional outcome were performed. Division of the groups according to, for instance, size, localization, type of tumour and level of amputation (e.g. above or below the knee) in some cases resulted in a relatively few number of patients in each subgroup. The risk of making errors of type I and II may thus be increased. For example, instead of studying the functional outcome of the whole group of patients, it could be argued that only patients with similar tumour locations in the two groups should be compared. However, the number of patients in each of these subgroups was too small to allow a meaningful comparison. Therefore, a different study including more patients and thus larger subgroups could produce different results. As a result of the above and the fact that the present study was a non-randomized study, the results are valid only for the patients included in this work and cannot directly be applied to another population of patients. On the other hand, the present study clearly indicates the importance of measuring functional outcome in future studies and especially in studies with an expected possible difference in functional outcome. Such a study with the necessary number of patients is in progress.

On the basis of the present results, we conclude that limb-salvage surgery is associated with a better functional outcome compared to amputation. Based on the Enneking system for functional evaluation also, the patients' general condition after limb-salvage is better compared to the patients' general condition after amputation. However, a more complex examination of the patients' quality of life is needed in order to conclude that limb-salvage is also associated with a better quality of life compared to amputation.

### Acknowledgements

The study was supported by the Clinical Research Unit, Danish Cancer Society, Oncologic Center, Aarhus University Hospital, Denmark.

### References

1 Enneking WF, Dunham W, Gebhardt MC, *et al.* A system for the functional evaluation of reconstructive procedures after surgical treatment of tumours of the musculoskeletal system. *Clin Orthop* 1993; 286:241–6.

2 Mouridsen HT, Dombernowsky P, Jensen OM, *et al.* Treatment of sarcomas in Denmark. *Ugeskrift for Lœger* 1995; 152:989–92.

3 Gustafson P, Dreinhofer KE, Rydholm A. Soft tissue sarcoma should be treated at a tumour center. A comparison of quality of surgery in 375 patients. *Acta Orthop Scand* 1994; 65:47–50.

4 Suit HD, Groeningen CJ, Mankin HJ, *et al.* Sarcoma of the soft tissues. In: Peckham M, Pinedo H, Veronesi U, eds. *Oxford textbook of oncology*. Oxford: Oxford University Press, 1995:1917–39.

5 Jensen OM, Kaae S, Madsen EH, *et al.* Histopathological grading in soft-tissue tumours. Relation to survival in 261 surgically treated patients. *Acta Pathol Microbiol Immunol Scand A* 1983; 91:145–50.

6 Enneking WF, Spanier SS, Goodmaan MA. Current concepts review. The surgical staging of musculoskeletal sarcoma. *J Bone Joint Surg Am* 1980; 62:1027–30.

7 Enneking WF, Spanier SS, Goodman MA. A system for the surgical staging of musculoskeletal sarcoma. *Clin Orthop* 1980; 153:106–20.

8 Karasek K, Constine LS, Rosier R. Sarcoma therapy: functional outcome and relationship to treatment parameter. *Int J Radiat Oncol Biol Phys* 1992; 24:651–6.

9 Chang AE, Steinberg SM, Culnana M, *et al.* Functional and psychosocial effects of multimodality limb-sparing therapy in patients with soft tissue sarcomas. *J Clin Oncol* 1989; 7:1217–28.

10 Bell RS, O'Sullivan B, Davis A, *et al.* Functional outcome in patients treated with surgery and irradiation for soft tissue tumours. *J Surg Oncol* 1991; 48:224–31.

11 Robinson MH, Spruce L, Eeles R, *et al.* Limb function following conservation treatment of adult soft tissue sarcoma. *Eur J Cancer* 1991 27:1567–74.

12 Lampert MH, Gerber LH, Glatstein E, *et al.* Soft tissue sarcoma: functional outcome after wide local excision and radiation therapy. *Arch Phys Med Rehabil* 1984; 65:477–80.

13 Stinson SF, DeLaney TF, Greenberg J, *et al.* Acute and long-term effects on limb function of combined modality limb sparing therapy for extremity soft tissue sarcoma. *Int J Radiat Oncol Biol Phys* 1991; 21:1493–9.

14 Rougraff BT, Simon MA, Greenberg DB, *et al.* Limb-salvage compared with amputation for osteosarcoma of the distal end of the femur. A long-term oncological, functional and quality-of-life study. *J Bone Joint Surg Am* 1994; 76:649–56.

15 Sugarbaker P, Barofsky I, Rosenberg SA, *et al.* Quality of life assessments of patients in extremity sarcoma clinical trials. *Surgery* 1982; 91:17–23.

16 Weddington WW, Segraves KB, Simon MA. Psychological outcome of extremity sarcoma survivor undergoing amputation or limb salvage. *J Clin Oncol* 1985; 3:1393–9.
